# Supraclavicular Approach of Lobectomy Improves Quality of Life for Patients With Unilateral Papillary Thyroid Microcarcinoma: A Prospective Cohort Study

**DOI:** 10.3389/fendo.2021.766444

**Published:** 2022-01-04

**Authors:** Shuai Xue, Qiuli Wang, Guang Chen, Peisong Wang, Li Zhang

**Affiliations:** ^1^ Department of Thyroid Surgery, The 1st Hospital of Jilin University, Changchun, China; ^2^ Department of Nephrology, The 1st Hospital of Jilin University, Changchun, China

**Keywords:** supraclavicular approach, lobectomy, papillary thyroid microcarcinoma, postoperative symptom, prospective

## Abstract

**Objective:**

Postoperative neck symptoms, including pain, swelling, uncomfortable feelings during swallowing, and incision adhesion formation, are common in patients after lobectomy through the traditional middle neck approach. A new unilateral supraclavicular approach is proposed to protect the anterior cervical region and reduce related complications. The aim of this study is to investigate the efficacy, safety, and advantages of the supraclavicular approach in lobectomy for unilateral papillary thyroid microcarcinoma (PTMC).

**Methods:**

Two hundred sixty-three patients were recruited into either a conventional middle group (CM) or a new supraclavicular (NS) group. Clinicopathological features, surgically related variables, and postoperative symptoms were recorded. Quality of life (QOL) of all patients was assessed by the 12-item short-form health survey (SF-12) and thyroid cancer-specific QOL (THYCA-QoL) questionnaire in 3 and 12 months.

**Results:**

There were no statistically significant differences in clinicopathological features (including sex, age, multifocality, extrathyroidal extension, histological variants, largest tumor diameter, Hashimoto’s thyroiditis, metastasized central lymph node, removed central lymph node, surgeon, BRAF mutation, and follow-up duration), hospitalization (including hospital cost, surgery time, and blood loss during surgery), and complications between the two groups. Patients who underwent lobectomy through the NS approach had significantly better SF-12 physical, mental, and THYCA-QoL than the CM group patients in both 3 and 12 months (all *p* < 0.001). Moreover, the NS group had a shorter hospitalization time.

**Conclusion:**

In conclusion, the NS approach for lobectomy is a safe and effective method for reducing postoperative symptoms and increasing QOL in patients with unilateral PTMC in both 3 and 12 months’ follow-up.

## Introduction

The incidence rate of thyroid cancer is increasing worldwide (1, 2). The rapid growth of papillary thyroid microcarcinoma (PTMC), which is defined as papillary thyroid carcinoma measuring ≤1 cm in the greatest dimension, is the main reason (3). The majority of PTMC patients have no clinical symptoms upon routine ultrasound (US) examination. In recent years, the development and popularization of high-frequency US and fine-needle aspiration (FNA) have further increased the diagnosis of PTMC (4). Most PTMC cases are low-risk. The recurrence rate is 1%–5%, and the 10-year mortality rate is as low as 0.3% (5). Therefore, active surveillance (AS) is recommended as an alternative treatment choice for low-risk PTMC cases (6). However, AS is not universally accepted because of the disparity in doctors’ knowledge and the concern of disease progression in the patient’s mind (7–9). Surgery is still the mainstay for the treatment of PTMC in many countries.

The good prognosis of PTMC led patients to pay more attention to postoperative quality of life (QOL), including postoperative functional recovery and cosmetic results (10). However, more than 50% of patients complain about a stretching, choking, or pressing feeling or discomfort or scarring in the middle neck postoperatively (11, 12). Some of them suffered these symptoms in the long term, which had a negative impact on QOL (13, 14). These neck discomforts might be caused by injury of the neck sensory nerve, adhesion of the larynx and strap muscles/subcutaneous tissues, and large tension of the middle neck, which is inevitable through the conventional middle (CM) neck approach (15, 16). Some studies have demonstrated better cosmetic results through the new supraclavicular (NS) approach (17, 18). But whether lobectomy through the NS approach could reduce neck discomforts and improve postoperative QOL is still unknown.

The aim of this study was to evaluate the potential benefits and risks of lobectomy through the NS approach. In this study, we will demonstrate several clinical concerns: 1) whether the NS approach reduces neck discomforts and improves neck postoperative QOL; and 2) whether the NS approach reduces postoperative complications. This study can demonstrate whether lobectomy through the NS approach is an effective and safe surgical method for unilateral PTMC.

## Methods

### Patient Selection

The Human Research Ethics Committee of the First Hospital of Jilin University approved this study (No. 2019-353). From November 2019 to July 2020, all unilateral PTMC patients who underwent lobectomy with unilateral central lymph node dissection (CLND) at the First Hospital of Jilin University were recommended for both CM and NS approaches at the same time. The surgeon will elaborate on both benefits and risks of these two surgical approaches, and patients can make a decision according to their preference. The exclusion criteria were as follows: patients’ age <18 years (1 case); those with surrounding neck structure invasion according to US and/or CT scan (6 cases); those with clinical lymph node metastasis (3 cases); those with persistent disease (0 cases); those with a history of neck radiotherapy (0 cases); and those with a history of previous thyroid surgery (3 cases). Finally, 263 unilateral PTMC patients were enrolled in our study.

### Diagnosis and Treatment

The majority of PTMC patients were detected by US examination and diagnosed by FNA and BRAF mutation detection. If the largest diameter of thyroid nodule was larger than 0.5 cm and presented with one or more US malignant features, FNA for suspicious thyroid nodule was recommended for patients according to the Chinese guidelines of PTMC (19). For those with suspicious nodules with a diameter <0.5 cm, AS was recommended, unless patients persisted on surgery because of concern about malignancy. CT scans were only performed for PTMC patients with potential surrounding tissue invasion. Lobectomy through CM approach was performed as follows: along the natural skin crease about two fingers above the sternal notch, a circular incision measuring 4 to 5 cm was performed. A subplatysmal flap was produced until the deep cervical fascia was exposed. After the strap muscle was divided and retracted in the center, the thyroid was exposed, and lobectomy was performed. The NS approach was performed: the incision was designed between the anterior and posterior sternocleidomastoid and two fingers above the clavicle. The space between the sternal and clavicular heads was separated, and the omohyoid muscle was identified. The omohyoid muscle was freed, then the space between the strap muscle and thyroid was separated. Lobectomy was performed on the CM group (**Supplementary 1 Video**; website: https://pan.baidu.com/s/1j7AHTLy_ws6-SlycRwX4nw; password: eo0t).

CLND was performed as previously described (20).

### Histopathological and Hospitalization Information

Histopathological characteristics, including multifocality, extrathyroidal extension (ETE), histological variants, largest tumor diameter (LTD), Hashimoto’s thyroiditis (HT), metastasized central lymph node (MCLN), removed central lymph node (RCLN), and BRAF mutation, were recorded. Hospitalization information and surgical complications like hospital stay, cost, surgical time, blood loss during surgery, hemorrhage, recurrent laryngeal nerve (RLN) injury, and parathyroid removal were also extracted from the patient’s database in the hospital. RLN injury was defined as loss of signal during neural monitoring and vocal cord paralysis during postoperative laparoscopy.

### Follow-Up and Quality of Life Evaluation

All patients were followed up with physical examinations, thyroid function, US, and QOL assessment at 3-month intervals. To assess QOL in 3 and 12 months, all enrolled PTMC patients completed two questionnaires: the 12-item short-form health survey (SF-12) and thyroid cancer-specific QOL (THYCA-QoL) questionnaire. The SF-12, which consists of 12 questions, is a well-validated short-form of SF-36. Physical and Mental Component Summary scores were calculated after answers were combined and weighted (**Supplementary 2** SF-12). Higher scores indicated a better health status. The THYCA-QoL contains 24 items that evaluate symptoms from thyroid cancer or its treatment, especially surgery. Each item was scored on a 4-point response scale ranging from 1, “not at all”, to 4, “very much”. Each score was converted into scores on seven multisymptom scales (neuromuscular, voice, concentration, sympathetic, throat/mouth, psychological, and sensory problems) and six single-symptom scales (problems with scarring, felt chilly, tingling hands/feet, gained weight, headache, and less interested in sex) (**Supplementary 3** THYCA-QOL). Scores were linear-transformed to a 0–100 scale, and a higher score on this scale indicates more complaints.

### Statistical Analysis

Continuous variables are presented as the means and standard deviations, while nominal variables are described as frequencies and proportions. The Mann–Whitney U test for continuous variables and Pearson’s chi-square tests were used for nominal variables, and *p* < 0.05 was considered statistically significant (2-sided). SPSS version 23 software (SPSS Inc., Chicago, IL) was used for all statistical analyses.

## Results

### Comparison of Baseline Characteristics

As shown in **Table 1**, baseline clinicopathological features, including sex, age, multifocality, ETE, histological variants, LTD, HT, MCLN, RCLN, surgeon, BRAF mutation, and follow-up duration, were compared between the CM and NS groups. These baseline characteristics, which may affect QOL, hospitalization, and surgical complications, were not different significantly between these two groups.

**Table 1:** Baseline clinicopathological feature comparison of PTMC patients between the CM and NS groups.

Variables	CM group: N = 108 (%)	NS group: N = 155 (%)	*p*-Value
Sex			
Female	100 (92.6)	143 (92.3)	
Male	8 (7.4)	12 (7.7)	0.92
Age, years	46.87 ± 10.38	46.45 ± 9.90	1.00
BMI, kg/m^2^	23.5 ± 8.2	22.1 ± 9.3	0.98
Multifocal tumor			
Yes	32 (29.6)	45 (29.0)	
No	76 (70.4)	110 (71.0)	0.92
ETE			
No	5 (4.6)	10 (6.5)	
Microscopic	102 (94.4)	144 (92.9)	
Gross	1 (1.0)	1 (0.6)	0.80
Histology variants			
Conventional	106 (98.1)	153 (98.7)	
Follicular	2 (1.9)	2 (1.3)	0.71
LTD (cm)	0.61 ± 0.21	0.54 ± 0.25	0.59
HT			
Yes	11 (10.2)	17 (11.0)	
No	97 (89.8)	138 (89.0)	0.84
MCLN	0.47 ± 1.05	0.51 ± 0.99	0.87
RCLN	3.81 ± 1.62	3.72 ± 1.66	0.91
Operator			
Prof. A	91 (84.3)	138 (89.0)	
Prof. B	17 (15.7)	17 (11.0)	0.26
BRAF mutation			
Yes	79 (73.1)	117 (75.5)	
No	4 (3.7)	9 (5.8)	
Unknown	25 (23.2)	29 (18.7)	0.54
Follow-up (months)	16.19 ± 2.87	16.57 ± 2.62	1.00

PTMC, papillary thyroid microcarcinoma; CM, conventional middle; NS, new supraclavicular; ETE, extrathyroidal extension; LTD, largest tumor diameter; HT, Hashimoto’s thyroiditis; MCLN, metastasized central lymph node; RCLN, removed central lymph node.

### Comparison of Overall Quality of Life, Hospitalization, and Complications

**Figure 1:**
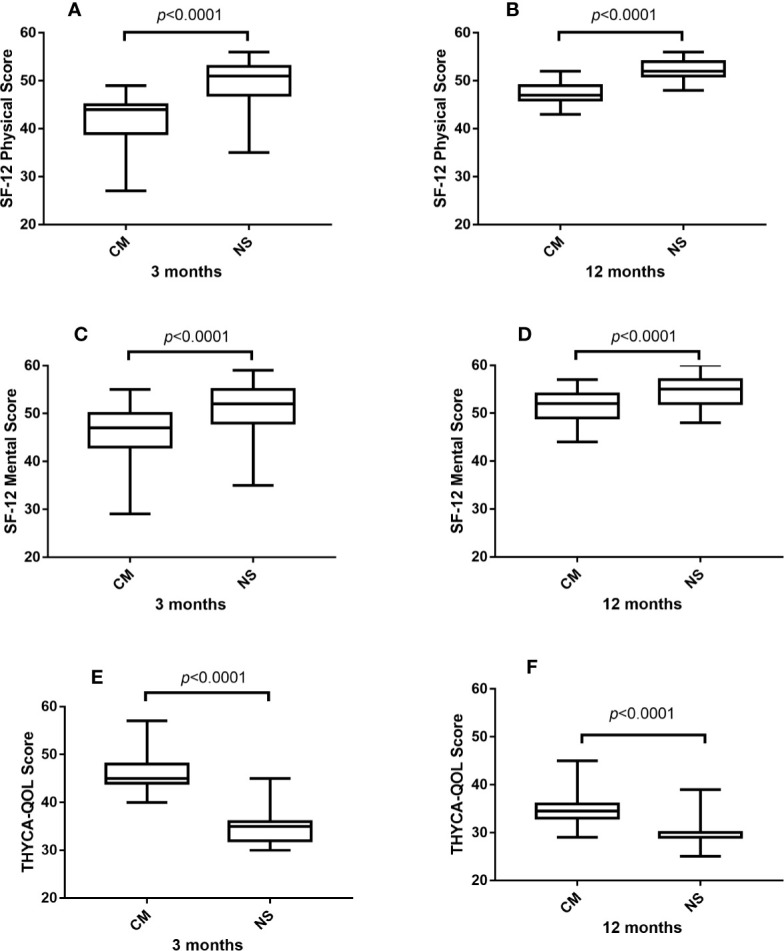
Comparison of SF-12 physical **(A, B)**, mental score **(C, D)**, and THYCA-QoL score **(E, F)** between CM and NS group in 3 and 12 months. SF-12, 12-item short-form health survey; THYCA-QoL, thyroid cancer-specific quality of life.

The comparison of general and thyroid cancer-specific QOL between CM and NS groups was performed and shown in **Figure 1**. Patients who underwent lobectomy through the NS approach had significantly better SF-12 physical, mental, and THYCA-QoL scores than the CM group patients in both 3 and 12 months (all *p* < 0.001). Moreover, hospitalization and surgical complications like hospital stay, cost, surgical time, blood loss during surgery, hemorrhage, RLN injury, and parathyroid removal were compared between these two groups. Hospital stay was significantly longer in the CM group (*p* = 0.04). Surgical complications related to the NS approach were low among both groups, and none of the complications were remarkably different between the groups, as shown in **Table 2**.

**Table 2:** Hospitalization and complications in PTMC patients.

Variables	CM group: N = 108 (%)	NS group: N = 155 (%)	*p*
Hospital stay (days)	5.21 ± 2.14	3.68 ± 0.75	0.04
Hospital cost (dollars)	3,064.76 ± 263.57	3,107.01 ± 211.25	0.88
Surgery time (min)	98.42 ± 13.30	104.11 ± 13.26	0.64
Blood loss during surgery (ml)	22.86 ± 4.96	23.17 ± 4.97	0.97
Hemorrhage			
Yes	0 (0)	1 (0.6)	
No	108 (100)	154 (99.4)	0.40
RLN injury			
Yes	1 (0.9)	4 (2.6)	
No	107 (99.1)	151 (97.4)	0.33
Parathyroid removal^#^			
Yes	2 (1.9)	5 (3.2)	
No	106 (98.1)	150 (96.8)	0.50

PTMC, papillary thyroid microcarcinoma; CM, conventional middle; NS, new supraclavicular; RLN, recurrent laryngeal nerve.

^#^Parathyroid removal: parathyroid was resected during surgery accidentally.

### Comparison of Postoperative Symptoms According to Thyroid Cancer-Specific Quality of Life

Postoperative symptoms, including neuromuscular, voice, concentration, sympathetic, throat/mouth, psychological, and sensory problems, problems with scarring, felt chilly, tingling hands/feet, gained weight, headache, and less interested in sex, were evaluated and recorded during follow-up (exclusion of possible concomitant pathologies like gastroesophageal reflux). As shown in **Table 3**, more patients of the CM group suffered severe postoperative negative symptoms such as neuromuscular, concentration, sympathetic, throat/mouth, psychological, and problems with scarring in both 3 and 12 months (all *p* < 0.05). On the other hand, patients of the NS group presented with fewer sensory problems only in 12 months (*p* < 0.05).

**Table 3:** THYCA-QoL comparison between the CM and NS groups.

THYCA-QoL	Months	CM (n = 108)	NS (n = 155)	*p*-Value
Neuromuscular	3	49.73 ± 13.48	43.33 ± 13.33	**0.03**
	12	43.21 ± 17.20	33.28 ± 14.40	**0.00**
Voice	3	37.84 ± 16.89	37.84 ± 13.41	1.00
	12	37.73 ± 11.86	37.45 ± 10.36	1.00
Concentration	3	46.03 ± 19.45	38.59 ± 12.71	**0.02**
	12	38.94 ± 14.00	31.45 ± 11.00	**0.00**
Sympathetic	3	49.72 ± 17.13	42.28 ± 19.44	**0.04**
	12	41.16 ± 14.12	34.79 ± 19.62	**0.02**
Throat/mouth	3	49.24 ± 21.60	31.3 ± 16.25	**0.00**
	12	44.4 ± 13.61	19.0 ± 11.52	**0.00**
Psychological	3	49.3 ± 20.46	34.86 ± 24.46	**0.00**
	12	40.96 ± 21.02	29.77 ± 12.40	**0.00**
Sensory	3	53.62 ± 12.33	49.18 ± 14.47	0.12
	12	48.23 ± 17.10	33.13 ± 21.10	**0.01**
Problems with scarring	3	58.3 ± 19.66	33.5 ± 15.68	**0.00**
	12	53.7 ± 20.54	18.4 ± 6.57	**0.00**
Felt chilly	3	42.69 ± 16.67	38.25 ± 15.44	0.21
	12	37.75 ± 17.11	35.75 ± 13.10	0.42
Tingling hands/feet	3	40.69 ± 12.44	36.25 ± 19.78	0.14
	12	36.45 ± 22.26	32.15 ± 19.26	0.10
Gained weight	3	37.94 ± 13.40	33.50 ± 14.72	0.08
	12	33.58 ± 15.22	28.42 ± 17.12	0.05
Headache	3	40.69 ± 12.56	36.25 ± 19.56	0.13
	12	37.25 ± 18.86	33.52 ± 15.66	0.09
Less interested in sex	3	42.99 ± 11.52	38.55 ± 11.27	0.07
	12	37.85 ± 19.20	34.98 ± 17.44	0.23

CM, conventional middle; NS, new supraclavicular; THYCA-QoL, thyroid cancer-specific quality of life.

Bold p-Values: p<0.05.

## Discussion

Still, until now, the potential benefits and risks of lobectomy through the NS approach for unilateral PTMC patients remain unknown. Our study demonstrated that patients who underwent lobectomy by the NS approach showed better physical, mental, and thyroid-cancer specific QOL than patients who underwent the CM approach after adjustment for all potential confounders for QOL. Moreover, patients in the NS group spent less time in the hospital, and they did not suffer significantly from more surgical complications. Furthermore, more patients of the CM group suffered severe postoperative negative symptoms such as neuromuscular, concentration, sympathetic, throat/mouth, and psychological problems with scarring in both 3 and 12 months.

The anterior neck area refers to the triangular area between the inner edges of the sternocleidomastoid muscles on both sides. The lower boundary is the sternal notch, while the upper boundary is the lower edge of the mandible. Moreover, the hyoid bone has divided this area into the upper and lower parts. And the lower part of the anterior neck area, which is also known as the anterior functional area of the neck, is the most vulnerable area affected by thyroid surgery (21–23).

The anterior area of the neck shares three main functions (24–27). 1) Sensory function: Lobectomy through the CM approach will injure the end branches of the transverse cervical nerve inevitably, which leads to skin numbness, pressing, or stitching feeling during wound healing. The NS approach will divide the space between the sternal and clavicular heads of the sternocleidomastoid muscle, which will not injure the transverse cervical nerve. 2) Movement function: Middle neck discomfort symptoms including difficulty in swallowing and pulling sensations during neck extension might be caused by adhesion of the larynx and strap muscles or subcutaneous tissues. Dysphagia was always reported after thyroid surgery through the CM approach because the adhesion restricts the upward movement of the larynx during swallowing. The NS approach will not divide the subplatysmal flap and strap muscle. And the adhesion between the larynx and other neck structures will not happen. 3) Cosmetic function: the tension of the middle neck is larger than that of the lateral neck. An incisional scar is more likely to form because of higher tension in the skin. The incision of the NS approach is located in the lateral neck and is more likely to get better cosmetic results. According to our study, the NS approach shared fewer postoperative neck negative symptoms (including neck discomforts, difficulty in swallowing, throat lump, and concerns about neck scarring) in both 3 and 12 months. The gasless transaxillary laparoscopic thyroid surgery also resects the thyroid between the sternal and clavicular heads of the sternocleidomastoid muscle. In agreement with our study, patients who underwent transaxillary laparoscopic thyroid surgery also presented with better postoperative neck symptoms (28–30).

However, lobectomy by the NS approach has some limitations: 1) removal of the contralateral gland, although feasible, is much more difficult through the NS approach. Excessive traction of the incision or separation of the strap muscle will increase the formation of postoperative incision scars. 2) Before surgery, evaluation of the location of the tumor and the lymph node metastasis in the central department is needed. If the tumor is more likely to invade the RLN, trachea, or esophagus, or the central lymph node metastasis is serious, it will be difficult to remove the contralateral gland. 3) If the tumor recurs in the contralateral gland, it is difficult to remove the contralateral gland again from the original incision.

Furthermore, several limitations in the study must be noted. First, the relatively small number of patients may generate some bias in our study. Also, the small number of patients limits the probability of propensity score matching to eliminate potential cofounders when the QOL between the CM group and NS group is compared. Some unknown and unmeasured confounders may influence the strength of the result, although many factors have been considered for baseline comparison, as shown in **Table 1**. Secondly, these clinical care strategies, like hospitalization time and cost for PTMC patients, were different from those in other countries. The potential discrepancy may limit the generalizability of these findings to other hospitals. Finally, different surgeons may have differences in incision protection, subcutaneous dissociation, and suture techniques. Ideally, it is best to compare patients of the same surgeon.

## Conclusion

In conclusion, lobectomy by the NS approach is a safe and effective method for reducing postoperative neck discomforts and increasing QOL in patients with unilateral PTMC in both 3 and 12 months’ follow-up.

## Data Availability Statement

The raw data supporting the conclusions of this article will be made available by the authors, without undue reservation.

## Ethics Statement

The studies involving human participants were reviewed and approved by the Human Research Ethics Committee of the First Hospital of Jilin University. The patients/participants provided their written informed consent to participate in this study.

## Author Contributions

All authors listed have made a substantial, direct, and intellectual contribution to the work and approved it for publication.

## Conflict of Interest

The authors declare that the research was conducted in the absence of any commercial or financial relationships that could be construed as a potential conflict of interest.

## Publisher’s Note

All claims expressed in this article are solely those of the authors and do not necessarily represent those of their affiliated organizations, or those of the publisher, the editors and the reviewers. Any product that may be evaluated in this article, or claim that may be made by its manufacturer, is not guaranteed or endorsed by the publisher.
