# Supplementary material for: Supraclavicular Approach of Lobectomy Improves Quality of Life for Patients With Unilateral Papillary Thyroid Microcarcinoma: A Prospective Cohort Study
Source: Front Endocrinol (Lausanne). 2022 Jan 4;12:766444. doi: 10.3389/fendo.2021.766444 (PMC8764192; doi:10.3389/fendo.2021.766444)
Supplement: Supplementary file 1 [file DataSheet_1.pdf]

SF-12® Patient Questionnaire

Patient Initials _____ _____ ______ **Date of Birth:** ___/___/___ **Patkey:** ______

Surgeon Name: ____ **Date:** ____________

Examination Period: ____ Postop 3 months ____ Postop 12 months

---

SF-12®:
This information will help your do ctors keep track of how you feel and how well you are able to do your usual activities. Answer every question by placing a check mark on the line in front of the appropriate answer. It is not specific for arthritis. If you are unsure about how to answer a question, please give the best answer you can and make a written comment beside your answer.

1. In general, would you say your health is:
   _____ Excellent (1)
   _____ Very Good (2)
   _____ Good (3)
   _____ Fair (4)
   _____ Poor (5)

The following two questions are about activities you might do during a typical day. Does YOUR HEALTH NOW LIMIT YOU in these activities? If so, how much?

2. MODERATE ACTIVITIES, such as moving a table, pushing a vacuum cleaner, bowling, or p laying golf:
   _____ Yes, Limited A Lot (1)
   _____ Yes, Limited A Little (2)
   _____ No, Not Limited At All (3)

3. Climbing SEVERAL flights of stairs:
   _____ Yes, Limited A Lot (1)
   _____ Yes, Limited A Little (2)
   _____ No, Not Limited At All (3)

During the PAST 4 WEEKS have you had any of the following problems with your work or other regular activities AS A RESULT OF YOUR PHYSICAL HEALTH?

4. ACCOMPLISHED LESS than you would like:
   _____ Yes (1)
   _____ No (2)

5. Were limited in the KIND of work o r other activities:
   _____ Yes (1)
   _____ No (2)

Surgeon Initials __________ **Date:** _____________

SF-12® Cont'd:

During the PAST 4 WEEKS, were you limited in the kind of work you do or other regular activities AS A RESULT OF ANY EMOTIONAL PROBLEMS (such as feeling depressed or anxious)?

6. ACCOMPLISHED LESS than you would like:
   _____ Yes (1)
   _____ No (2)

7. Didn't do work or other activities as CAREFULLY as usual:
   _____ Yes (1)
   _____ No (2)

8. During the PAST 4 WEEKS, how much did PAIN interfere with your normal work (including both work outside the home and housework)?
   _____ Not At All (1)
   _____ A Little Bit (2)
   _____ Moderately (3)
   _____ Quite A Bit (4)
   _____ Extremely (5)

The next three questions are about how you feel and how things have been DURING THE PAST 4 WEEKS. For each question, please give the one answer that comes closest to the way you have been feeling. How much of the time during the PAST 4 WEEKS –

9. Have you felt calm and peaceful?
   _____ All of the Time (1)
   _____ Most of the Time (2)
   _____ A Good Bit of the Time (3)
   _____ Some of the Time (4)
   _____ A Little of the Time (5)
   _____ None of the Time (6)

Surgeon Initials __________ Date: _____________

SF-12® Cont'd:

---

10. Did you have a lot of energy?
    _____ All of the Time (1)
    _____ Most of the Time (2)
    _____ A Good Bit of the Time (3)
    _____ Some of the Time (4)
    _____ A Little of the Time (5)
    _____ None of the Time (6)

11. Have you felt downhearted and blue?
    _____ All of the Time (1)
    _____ Most of the Time (2)
    _____ A Good Bit of the Time (3)
    _____ Some of the Time (4)
    _____ A Little of the Time (5)
    _____ None of the Time (6)

12. During the PAST 4 WEEKS, how much of the time has your PHYSICAL HEALTH O R EMOTIONAL PROBLEMS interfered with your social activities (like visiting with friends, relatives, etc.)?
    _____ All of the Time (1)
    _____ Most of the Time (2)
    _____ A Good Bit of the Time (3)
    _____ Some of the Time (4)
    _____ A Little of the Time (5)
    _____ None of the Time (6)

Surgeon Signature____ **Date**___________________________

SF-12® Health Survey © 1994, 2002 by Medical Outcomes Trust and QualityMetric Incorporated. All Rights Reserved
SF-12® is a registered trademark of Medical Outcomes Trust
