# Supplementary material for: Supraclavicular Approach of Lobectomy Improves Quality of Life for Patients With Unilateral Papillary Thyroid Microcarcinoma: A Prospective Cohort Study
Source: Front Endocrinol (Lausanne). 2022 Jan 4;12:766444. doi: 10.3389/fendo.2021.766444 (PMC8764192; doi:10.3389/fendo.2021.766444)
Supplement: Supplementary file 2 [file DataSheet_2.pdf]

THYCA-QOL **Questionnaire**

Patient Initials ______ ______ _______Date of Birth: _____/_____/_____Patkey: _______
Surgeon Name: ___________________ Date: _________________
Examination Period: ______ Postop 3 months ______ Postop 12 months

Final 24 items of the questionnaire

1. Have you had a dry mouth?
2. Have you had trouble swallowing?
3. Have you been hoarse?
4. Have you had a weak voice?
5. Have you had a lump in your throat?
6. Did the scar in your neck bother you?
7. Have you felt chilly?
8. Have you had difficulty tolerating heat?
9. Have you had hot flushes?
10. Have you had pain in your muscles and/or joints?
11. Have you had tingling hands and/or feet?
12. Have you had cramps in your legs?
13. Have you felt slowed down?
14. Have you gained weight?
15. Have you had painful, irritated or dry eyes?
16. Have you had skin problems (e.g. itchy, dry)?
17. Have you suffered from palpitations?
18. Have you had headaches?
19. Have you had abrupt attacks from tiredness?
20. Have you had troubles with thinking?
21. Have you had attention problems?
22. Have you felt restless or agitated?
23. Have you felt anxious?
24. To what extent were you interested in sex?

1, Not at all; 2, A little; 3, Quite a bit; 4, Very much
